# Data-Driven Design of Transparent Thermal Insulating Nanoscale Layered Oxides

**DOI:** 10.3390/mi14010186

**Published:** 2023-01-11

**Authors:** Yen-Ju Wu, Yibin Xu

**Affiliations:** 1 Research and Service Division of Materials Data and Integrated System (MaDIS), National Institute for Materials Science (NIMS) 1-1 Namiki, Tsukuba 305-0044, Ibaraki, Japan; 2 International Center for Young Scientists (ICYS), National Institute for Materials Science (NIMS), 1-2-1 Sengen, Tsukuba 305-0047, Ibaraki, Japan

**Keywords:** thermal conductivity, thermal insulator, thin film, superlattice, transparency

## Abstract

Predicting the interfacial thermal resistance (ITR) for various material systems is a time-consuming process. In this study, we applied our previously proposed ITR machine learning models to discover the material systems that satisfy both high transparency and low thermal conductivity. The selected material system of TiO_2_/SiO_2_ shows a high ITR of 26.56 m^2^K/GW, which is in good agreement with the predicted value. The nanoscale layered TiO_2_/SiO_2_ thin films synthesized by sputtering exhibits ultralow thermal conductivity (0.21 W/mK) and high transparency (>90%, 380–800 nm). The reduction of the thermal conductivity is achieved by the high density of the interfaces with a high ITR rather than the change of the intrinsic thermal conductivity. The thermal conductivity of TiO_2_ is observed to be 1.56 W/mK with the film thickness in the range of 5–50 nm. Furthermore, the strong substrate dependence is confirmed as the thermal conductivity of the nanoscale layered TiO_2_/SiO_2_ thin films on quartz glass is three times lower than that on Si. The proposed TiO_2_/SiO_2_ composites have higher transparency and robustness, good adaptivity to electronics, and lower cost than the current transparent thermal insulating materials such as aerogels and polypropylene. The good agreement of the experimental ITR with the prediction and the low thermal conductivity of the layered thin films promise this strategy has great potential for accelerating the development of transparent thermal insulators.

## 1. Introduction

Transparent thermal insulating materials have been widely used in decreasing heat loss, collecting solar energy, and increasing efficiency of clean energy usage, such as thermal collectors [1], phase-change memory that relies on self-heating to switch between memory states [2], thermoelectrics [3], and thermal insulation windows [4]. For such applications, low thermal conductivity and high transmittance are the two essential properties. The ability to reduce heat loss and to provide high transmittance depends on the material and operating temperature. Porous materials such as aerogels, polymer materials such as polypropylene, and amorphous SiO_2_ (a-SiO_2_) have been conventionally used as transparent thermal insulators. More specifically, aerogels have been frequently used to provide good thermal insulation. However, there are still issues to be overcome for those materials. For example, ensuring good thermal insulation and the high transparency of silica aerogels still remains to be a big challenge [5]. For porous materials, their low robustness and high cost limit their application. For polymer materials, although they generally show lower thermal conductivity and relatively high transmittance of ~80%, their low melting points reduce its practicality at high temperatures. As for inorganic a-SiO_2_, although it is commonly used as a thermal insulator in electronics, its thermal conductivity (1.38 W/mK) [6] is higher than that of aerogels or polymers.

On the other hand, inorganic nanocomposite structures with periodic or aperiodic multilayers have become new prominent candidates to reduce thermal conductivity to a value that is even lower than that of homogeneous amorphous structures. Cahill et al. found that the Si–Ge superlattice exhibited a large reduction in its thermal conductivity because of the interfacial thermal resistance (ITR) [7]. The larger interface density was proven to contribute to the lower thermal conductivity compared to the continuous films in various nanolaminates [8,9,10,11,12]. Hu et al. proposed the aperiodic layered structures of graphene and MoS_2_ to reduce thermal conductivity [13]. In such structures, it is considered that the phonon propagation is hindered by scattering into random directions or associated interferences when phonons encounter interfaces in nanostructured materials.

So far, various methods to describe the ITR at the interface are proposed such as the acoustic mismatch model (AMM) [14], the diffuse mismatch model (DMM) [15], and the molecular dynamics (MD) simulation [16,17,18]. Although these models and simulations provide a theoretical way to evaluate the ITR, it is still difficult to achieve high accuracy or to conduct high-throughput predictions. Although AMM and DMM provide simple pictures on the ITR, many factors are overlooked which may affect the ITR in those models. MD simulations can include those factors, but their computational cost is generally high and predicting the ITR with high accuracy would be a time-consuming process. Another approach to predict the ITR is machine learning and we have shown this approach in our previous work [19]. Once we trained a machine learning model, making predictions is a significantly time-efficient process compared to the conventional methods. By utilizing machine learning and the databases for the ITR and other materials properties, we can construct a model that takes into account various chemical, physical, and synthesis process factors to predict the ITR with high accuracy [19,20]. In our previous studies, we have successfully realized the nanocomposite thin films with ultralow thermal conductivity by the interfacial design based on machine learning [19,21]. In this study, we further discuss how we consider the transparent property through the combination of machine learning and experimental optimization to realize the transparent thermal insulators based on the ITR machine learning model constructed in our previous works.

High optical transmittance is another crucial issue to be addressed in relation to transparent thermal insulating materials. To discover material systems that satisfy both properties, the search space for material candidates should be confined to transparent materials with large band gaps. We selected transparent material systems with high ITR based on the prediction by the ITR model. Further, we synthesized those selected material systems through the nanocomposite optimization by sputtering and measuring their ITR and optical transmittance. The thermal conductivity and the ITR were measured by the frequency-domain thermoreflectance (FDTR). The structures of the layered films with various periodic thicknesses and substrates were characterized by X-ray diffraction (XRD) and transmission electron microscopy (TEM).

## 2. Experimental Procedure

### 2.1. Thin Film Deposition

The TiO_2_/SiO_2_ layered thin film samples were prepared on quartz glass (Qz) or Si substrates in the sputtering system (CFS-4EP-LL, Shibaura Mechatronics Corp., Yokohama, Japan) at a pressure of approximately 6 × 10^−5^ Pa before deposition. The pressure was maintained at 0.4 Pa (20 sccm Ar flow) during the deposition process. Ar was used as the sputtering gas for Au at 20 sccm, whereas both Ar and O_2_ were applied for TiO_2_ (Ar: 16 sccm; O_2_: 4 sccm) and SiO_2_ (Ar: 13 sccm; O_2_: 13 sccm)_._ The RF power for both TiO_2_ and SiO_2_ was set at 200 W, while the DC power was set at 50 W for Au. TiO_2_:SiO_2_ in Table 1 shows the thicknesses of TiO_2_ and SiO_2_ layers corresponding to the quartz crystal resonator. The thicknesses of TiO_2_ and SiO_2_ were set to be 1 nm, 5 nm, and 30 nm. The 30 nm sample (total thickness is 60 nm) is used to validate the ITR with the predicted values, and the 1 nm and 5 nm samples (total thickness is 100 nm) are used for analyzing the interface effect on the thermal conductivity. After the deposition of the TiO_2_/SiO_2_, an Au layer with the thickness of 120 nm was deposited without evacuation at the top as a heat absorber for the thermal measurement. The total film thickness and the thickness of each layer were analyzed through TEM (JEM-ARM200F, JEOL Ltd., Tokyo, Japan). The structural properties of the thin film were characterized through XRD (SmartLab, Rigaku Corp., Tokyo, Japan).

### 2.2. Heat Conduction Equation for the ITR and Thermal Conductivity

The thermal resistance measurement was performed through FDTR [22]. The thermal resistance was measured along the perpendicular direction (cross-plane) to the Qz or Si substrate. Heat conduction was assumed to be one-dimensional because the laser spot was much larger than the film thickness (Equation (1)) [23].

(1) $$\frac{T\left(0\right)}{qd_{0}}=\frac{e^{-i\frac{\pi }{4}}}{\sqrt{2\omega \lambda_{3}C_{3}}}+R_{0}+\left(1-\frac{\lambda_{2}C_{2}}{\lambda_{3}C_{3}}\right)\frac{d_{2}}{\lambda_{2}}+\left(1-\frac{\lambda_{1}C_{1}}{\lambda_{3}C_{3}}\right)\frac{d_{1}}{\lambda_{1}}+\left(\frac{1}{2}-\frac{\lambda_{0}C_{0}}{\lambda_{3}C_{3}}\right)\frac{d_{0}}{\lambda_{0}}$$

where $T\left(0\right)$ is the Au temperature; q is heat per unit volume; C is heat capacity per unit volume; λ is thermal conductivity; $R_{0}$ is the sum of the ITRs at Au/SiO_2_, TiO_2_/SiO_2_ (it varies with the interface number), and TiO_2_/substrate; subscripts 0, 1, 2, and 3 denote Au, TiO_2_, SiO_2_, and the substrate, respectively. The temperature on the Au film surface, $T\left(0\right)$, was detected through the thermoreflectance method using a probe laser with the applied alternating current with the frequency ω. If we plot $\frac{T\left(0\right)}{qd_{0}}$ versus $\omega^{-1/2}$, the intercept *R* gives the sum of the last four terms of Equation (1). The second term, $R_{0}$, can be calculated with the known thickness, specific heat, and thermal conductivity of the Au, SiO_2_, TiO_2_ films, and the substrate. We also conducted the measurement for the TiO_2_ films with different thicknesses and plotted *R* as a function of *d*. The slope of the line was equal to $\frac{1}{\lambda_{1}}-\frac{C_{1}}{\lambda_{3}C_{3}}$. The thermal conductivity of TiO_2_ ($\lambda_{1}$) was obtained as 1.56 W/mK at all the thicknesses we measured: 5 nm, 10 nm, 30 nm, and 50 nm. The value was lower than the reported value of bulk polycrystalline TiO_2_ (8.9 W/mK) [24]. Table 2 presents the thermophysical properties of the materials used in the calculation. We subsequently determined the thermal conductivity along the cross-plane by dividing the film thickness by the total thermal resistance.

## 3. Result

### 3.1. Data-Driven Material Selection

**Figure 1:**
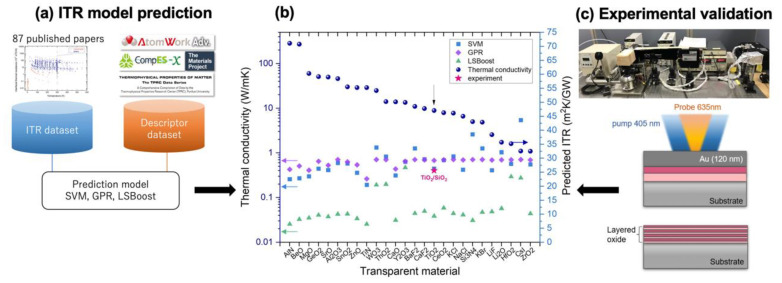
Schematics of the data-driven material selection to combine the machine-learning method and experimental validation. (**a**) ITR prediction model. Detailed information of the datasets is presented in our previous work [26]. (**b**) Thermal conductivity of various transparent materials and their predicted ITR with SiO_2_. (**c**) Schematic of the experimental validation set up. The images show the FDTR of ITR measurement. The probe laser of 635 nm and pump laser of 405 nm were applied for detecting and heating the sample. The bilayer thin film with Au-top layer deposited on the substrate was used for evaluating the ITR value after the prediction with the model. TiO_2_/SiO_2_ with a high ITR of 26.56 m^2^K/GW (pink star in (**b**)), which was close to the predicted values, was selected.

First, the proposed ITR machine learning model [19] was applied to select the transparent interface with high ITR. Figure 1 shows the flow chart of our data-driven scheme for the materials selection. We used ITR dataset, which was collected from 87 published papers, and the descriptor dataset constructed for 298 single-element materials or binary compounds. The ITR dataset contains the ITR values of various interfaces with temperature, the synthesis method, the thermal measurement method, the sample pretreatment, and its original references. The descriptor dataset is composed of the physical (e.g., melting point, density), chemical (e.g., electronegativity, binding energy), and process (e.g., film thickness) descriptors. The details of the datasets are available in our previous work [26].

According to our previous works, ordinal least-square linear regression showed poor predictive performance and least absolute shrinkage and selection operator (LASSO) neglected important descriptors such as measurement temperature [19,26]. Support vector machine regression (SVM), Gaussian process regression (GPR), and LSBoost regression were used in our previous studies in consideration of the dataset size and their ability to describe nonlinear effects. SVM and GPR are kernel-based methods and radial basis function (RBF) kernel was used for both methods. LSBoost regression performs least-square boosting which fits regression ensembles to minimize mean-squared error. The coefficient of determination, *R*^2^, of SVM, GPR, and LSBoost are 0.879, 0.916, and 0.919, respectively. The initial algorithm settings and additional details on those algorithms are described in our previous papers [19,20].

The transparent materials in the searching space were screened by the band gap that was >2.8 eV to be transparent in the visible range. Note that some transparent materials might be excluded due to the underestimation of the simulated values for band gap [27]. The 70 materials, which met the band gap criterion and had all the necessary descriptors for the ITR prediction, formed more than 4800 possible candidate interfaces. To reduce the number of candidates, one of the materials in the interface was fixed as SiO_2_ due to its easiness to synthesize and its low thermal conductivity. The ITR values predicted by our machine learning models and experimental thermal conductivity values in the polycrystalline bulk form, which were collected from Thermophysical Properties Research Center Data Series [24], are shown in Figure 1b. The ITR values predicted by SVM, GPR, and LSBoost models are shown by the blue, purple and green colors, respectively. Generally, the predicted values differ among various models even if the coefficient of determination of *R*^2^ is all higher than 0.85. Therefore, we selected the candidates that were predicted to have high ITR by all the three models. The experimental ITR of TiO_2_/SiO_2_ (TS-Qz-30 sample), 26.56 m^2^K/GW, was close to the predicted values (Figure 1b). After the experimental validation of the ITR of TiO_2_/SiO_2_, we further synthesized TiO_2_/SiO_2_ into nanoscale layered thin films (Figure 1c) by sputtering to increase the interface density and analyze the ITR effect on the thermal conductivity.

### 3.2. Structure

**Figure 2:**
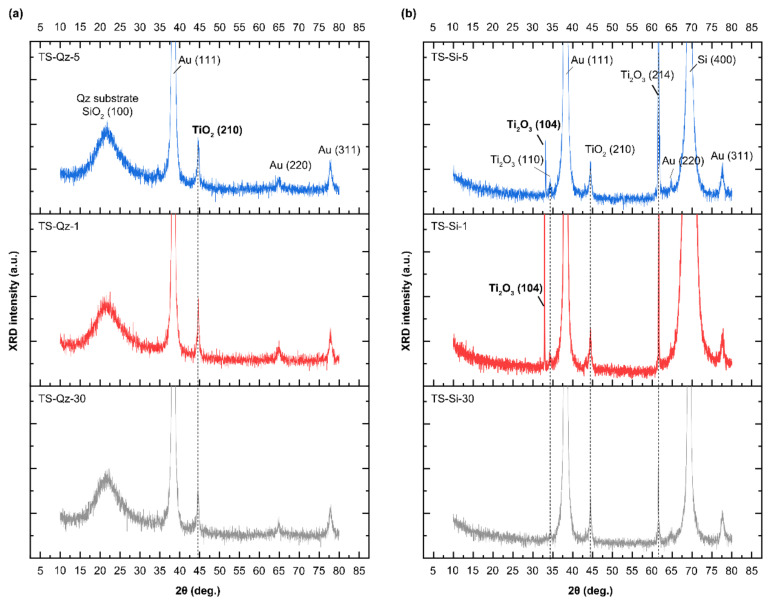
X-ray diffraction (XRD) of TiO_2_/SiO_2_ on the quartz glass (Qz) (**a**) and Si (**b**) substrates.

Figure 2 shows the XRD pattern of the TiO_2_/SiO_2_ samples. The samples on both the Qz and Si substrates showed rutile TiO_2_(210) and Au phases of the top layer, indicating that the films were composed of crystalline TiO_2_ and amorphous SiO_2_. The SiO_2_(100) peak came from the Qz substrate instead of the layered thin film. Interestingly, the additional phases of Ti_2_O_3_ (104), (110), and (214) only existed in the samples on the Si substrates, although the samples with the same layer thickness on Qz and Si were simultaneously deposited in the same sputtering. The existence of these additional phases may be attributed to the same tetrahedral atomic environment of O in Ti_2_O_3_ and of Si in Si and the similar atomic distance between O–Ti (0.203 nm) in Ti_2_O_3_ and Si–Si (0.235 nm) in Si. Moreover, the peak intensity of Ti_2_O_3_ (104) increased with the number of interfaces, implying the strong relation between formation of the Ti_2_O_3_ phase and the interfacial region.

**Figure 3:**
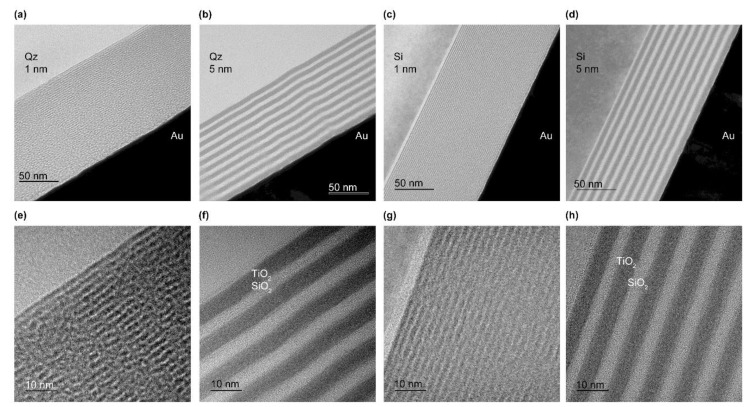
TEM images of the TiO_2_/SiO_2_ multilayered samples. Samples on quartz glass (Qz) of (**a**,**e**) 1 nm and (**b**,**f**) 5 nm for each layer and on Si of (**c**,**g**) 1 nm and (**d**,**h**) 5 nm for each layer. The total thickness of all samples was 100 nm. The scale bar is 50 nm and 10 nm for upper and bottom, respectively.

Figure 3 presents TEM images of the layered samples on the Qz and Si substrates. The experimental parameters are shown in Table 1 for samples of (a,e) TS-Qz-1, (b,f) TS-Qz-5, (c,g) TS-Si-1, and (d,h) TS-Si-5. All films were deposited well without porosities. The thickness of each layer matched the sputtering deposition setting of 1 and 5 nm. The interface of the thinner-layer samples (Figure 3e,g) was unclear when compared with the thicker-layer samples (Figure 3f,h).

### 3.3. Transmittance

**Figure 4:**
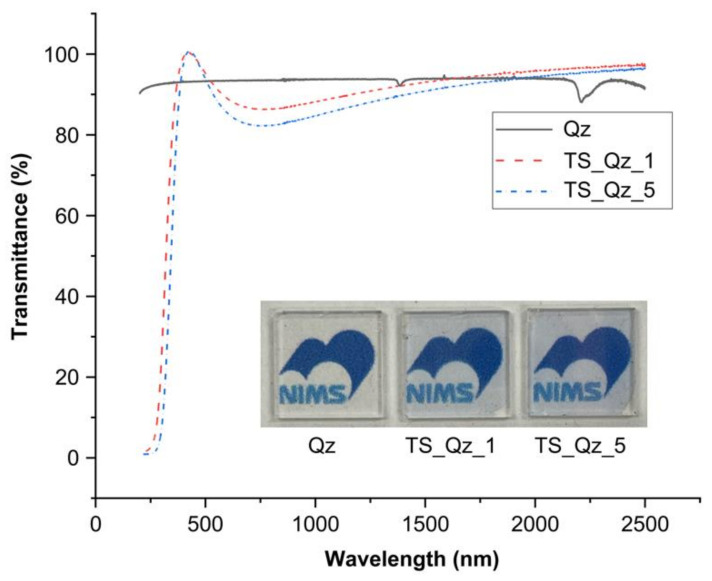
The transmittance of the nanoscale layered TiO_2_/SiO_2._ Inset: images of the corresponding films.

Figure 4 shows that the nanoscale layered TiO_2_/SiO_2_ has high transmittance in the visible range of 380–800 nm. It shows higher transmittance in the range of 380–500 nm while lower transmittance in the range of 550–800 nm compared to Qz. The average transmittance in the visible range of the nanoscale layered TiO_2_/SiO_2_ reached 92.6% and 91.1% for TS_Qz_1 and TS_Qz_5, respectively.

### 3.4. ITR and Thermal Conductivity at Room Temperature

Table 3 presents the cross-plane thermal conductivity ($\lambda_{⊥}$) of the samples with various number of interface (*N*). *R*_0_*, which is obtained by subtracting the ITR of Au/SiO_2_ (5 m^2^K/GW) [6] from *R*_0_ (the second term in Equation (1)), is the ITR of all the TiO_2_/SiO_2_ interfaces. The ITR and thermal conductivity were measured by FDTR and evaluated via Equation (1) with the thermophysical properties in Table 2. Detailed information is shown in Section 2.2.

The thermal conductivity of the samples on Qz decreased from 0.26 W/mK to 0.21 W/mK and that of the samples on Si decreased from 0.96 W/mK to 0.54 W/mK with the increasing interfaces (*N*: 20 to 100). All samples deposited on Qz showed lower thermal conductivities than those on the Si substrates. The ITR of each interface (*R*_0_*/*N*) decreased as the thickness of each layer decreased.

## 4. Discussion

### 4.1. ITR

The thermal conductivity and the ITR of all samples with different layer thicknesses were measured and calculated, as described in Section 3.4. The TiO_2_/SiO_2_ samples on Qz and Si both exhibited a decrease in the thermal conductivity compared with the respective bulk constituents of SiO_2_ and TiO_2_. Their thermal conductivities at 300 K decreased by 85% from SiO_2_ and by 87% from TiO_2_.

If we use the thermal resistor model reported by Böttger et al. [28] in Equation (2) to predict the thermal conductivity ($\lambda_{⊥}$), which is in the case of negligible interface resistance, we obtain:

(2) $$\lambda_{⊥}=\lambda_{2}\frac{\lambda_{1}/\lambda_{2}\left(1+d_{1}/d_{2}\right)}{d_{1}/d_{2}+\lambda_{1}/\lambda_{2}}$$

where λ represents thermal conductivity; d represents thickness; and subscripts 1 and 2 represent the material beside the interface. The estimated thermal conductivity of the multilayered TiO_2_/SiO_2_ of 1.46 W/mK was higher than our observation in Table 3. The overestimation of $\lambda_{⊥}$ indicated that the ITR contribution cannot be neglected.

### 4.2. Structural Effect on the ITR

The ITR decreased as the thickness of each layer decreased from 25.64 m^2^K/W (30 nm, Qz) to 4.08 m^2^K/W (1 nm, Qz) (Table 3). The structure of the interfacial region approached the amorphous state as the thickness of each layer decreased. The phonon modes available for heat transfer were then broadened [29]. This may cause more overlapping phonon modes, in which heat can be transported between two materials beside the interfaces, resulting in a lower ITR. Moreover, with the increase of interface density, the layer thickness became thinner than the mean free path of the dominant phonons. The phonons may have a high possibility to transport (tunnel) across the interfaces and attribute to the lower thermal resistance at the interfaces [11]. In short, the thickness-dependent ITR is significant for the nanoscale layered TiO_2_/SiO_2_. All samples deposited on Qz (without Ti_2_O_3_ phases) showed lower thermal conductivities relative to those on the Si substrates (with Ti_2_O_3_ phases), corresponding to Figure 2. This may imply the low ITR of Ti_2_O_3_/SiO_2_ relative to TiO_2_/SiO_2_.

To summarize the structural effect of the nanoscale layered TiO_2_/SiO_2_ on the ITR, the following three are preferred to realize low thermal conductivity (high ITR): (1) samples deposited on Qz to prevent the Ti_2_O_3_ phase formation; (2) sharp interface; and (3) high interface density. A trade-off existed between the film thickness and the interface density when the total thickness was fixed. The aperiodic stacking order can disrupt the secondary periodicity (superlattice phonons) and reduce the phonon lifetimes, resulting in lower thermal conductivity. Therefore, the stacking order can be further optimized by combining it with the proposed strategy of material system selection.

### 4.3. Comparison with Other Transparent Layered Materials

**Figure 5:**
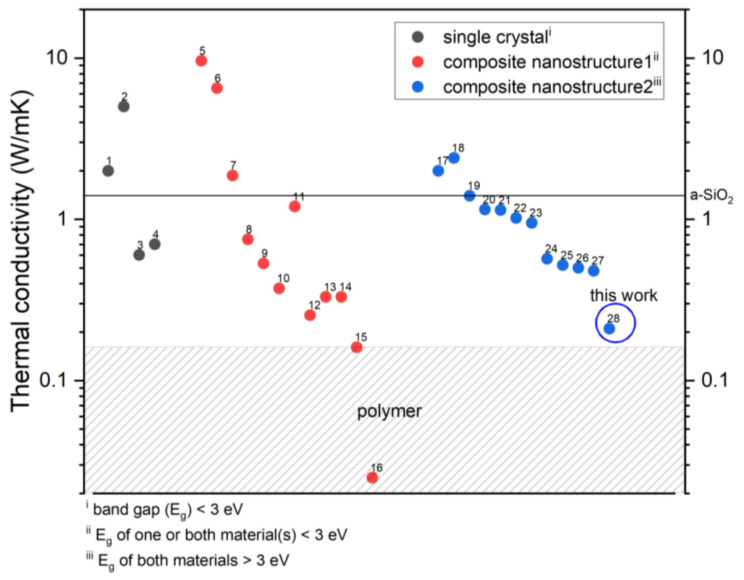
Thermal conductivity at R.T. of the reported inorganic pore-free materials. The label corresponds to the material systems in Table 4. Eg > 3 eV (<3 eV), the material is transparent (nontransparent) to visible light. In this study, TiO_2_/SiO_2_ achieved the lowest thermal conductivity compared to the other reported transparent layered composites.

Table 4 and Figure 5 show the thermal conductivities of the reported inorganic pore-free materials ranging from 0.48 W/mK to 18.0 W/mK. The materials were categorized into single crystals and composite nanostructures. The composite nanostructures were classified by the band gap value (*E_g_*) of 3 eV. If the *E_g_* of at least one material of the composite nanostructure is smaller than 3 eV (composite nanostructure 1), it is considered to be nontransparent. If the *E_g_* of both materials are larger than 3 eV (composite nanostructure 2), it is considered to be transparent. Most of the composite nanostructures reach a lower thermal conductivity than the single crystal (e.g., PbTe [30], Zn_4_Sb_3_ [31], SnSe [32], and AgSbTe_2_ [33]). In our previous work, Bi/Si reached an ultralow thermal conductivity of 0.16 W/mK [21], which is as low as that of polymer materials. Hu et al. proposed an aperiodic stacking composite nanostructure of graphene/MoS_2_ with an even lower thermal conductivity [13]. The abovementioned thermal insulators are unsuitable for transparent applications because of the smaller *E_g_* of the materials in the nanostructure. In comparison with the reported composite nanostructures composed of the materials with *E_g_* > 3.0 eV, the thermal conductivity of the layered TiO_2_/SiO_2_ in this study achieves the lowest values of 0.21 W/mK as shown in Figure 5. The characteristics of lower cost and higher robustness than porous materials (e.g., aerogel), the higher temperature tolerance than polymers, and lower thermal conductivity than the a-SiO_2_ (common thermal insulators in electronics) promise the nanoscale layered TiO_2_/SiO_2_ as transparent thermal insulators for thermal collectors, thermal insulation windows, or thin film thermoelectric.

## 5. Summary

A data-driven strategy for exploring inorganic material systems with high transparency and high ITR for the transparent thermal insulator was demonstrated. We applied an ITR machine learning model, which can consider the chemical, physical, and process properties for predicting ITR and achieve higher predictive performance than the conventional prediction models (AMM, DMM), to select the transparent material systems among more than 4800 candidates. After the experimental validation, TiO_2_/SiO_2_ was selected in terms of easiness of synthesis and the intrinsic low thermal conductivity. This material system achieved a high ITR of 26.56 m^2^K/GW, which is in good agreement with the prediction by our machine learning model. Through the ITR prediction by machine learning, the exploration of the material systems for transparent thermal insulators can be accelerated.

The TiO_2_/SiO_2_ nanoscale layered thin films with high interface density were further synthesized by sputtering. They showed an ultralow thermal conductivity of 0.21 W/mK and high transmittance to visible light. The low thermal conductivity was attributed to the high ITR between the alternating layers and the low intrinsic thermal conductivity of the component materials. The layered thin films on the quartz substrate had lower thermal conductivity than those on Si due to the substrate dependence of the structure.

The good agreement of the experimental ITR with the prediction and the low thermal conductivity of the layered thin films promise that this strategy has great potential for developing transparent thermal insulators in the future. Note that the compound formation at the interfaces and the substrate dependence, which are yet considered in material searching, also have a substantial effect on the ITR. An improvement made by further considering experimental parameters and interfacial reactions would give a more comprehensive exploration.

## Figures and Tables

**Table 1:** Experimental parameters of the TiO_2_/SiO_2_ samples.

Sample	TiO_2_:SiO_2_ [nm]	The Number of Interfaces	Substrate
TS-Qz-30	30:30	2	Qz
TS-Qz-5	5:5	20	Qz
TS-Qz-1	1:1	100	Qz
TS-Si-30	30:30	2	Si
TS-Si-5	5:5	20	Si
TS-Si-1	1:1	100	Si

**Table 2:** Thermophysical properties of the materials used in the calculation.

Material	Volumetric Heat Capacity (×10^6^ J/m^3^K)	Thermal Conductivity (W/mK)	ITR (m^2^K/GW)
Au	2.509 [24,25]	298 [24,25]	-
TiO_2_	2.76 [24,25]	1.56	-
Si	1.66 [24,25]	148 [24]	-
SiO_2_	1.65 [24,25]	1.38 [6,24]	-
Au/SiO_2_			5 [6]
Au/TiO_2_ and TiO_2_/Qz			23.26

**Table 3:** Thermal conductivity of the samples. *N* is the number of the interfaces.

Sample	*N*	$\lambda_{⊥}(W/\mathrm{mK})$	*R*_0_*/*N* (m^2^K/GW)
TS-Qz-30	2	0.65	25.64
TS-Qz-5	20	0.26	15.71
TS-Qz-1	100	0.21	4.08
TS-Si-30	2	0.97	10.38
TS-Si-5	20	0.96	1.82
TS-Si-1	100	0.54	1.18

**Table 4:** Thermal conductivity at room temperature (R.T.) of the reported inorganic pore-free materials.

ID	Material	Thermal Conductivity (W/mK)	Ref.
1	PbTe	2	[30]
2	Zn_4_Sb_3_	5	[31]
3	SnSe	0.6	[32]
4	AgSbTe_2_	0.7	[33]
5	Si/SiGe	9.6	[34]
6	GaAs/AlAs	6.5	[35]
7	Si/Ge SLNWs (superlattice nanowires)	1.87	[36]
8	Si/SiO_2_	0.75	[37]
9	W/Al_2_O_3_	0.53	[38]
10	Ta/TaOx	0.37	[39]
11	Mo/Si	1.2	[40]
12	Ge_2_Sb_2_Te_5_/ZnS:SiO_2_	0.25	[41]
13	Au/Si	0.33	[42]
14	Bi_2_Te_3_/Sb_2_Te_3_	0.33	[43]
15	Bi/Si (previous work)	0.16	[21]
16	graphene/MoS_2_	0.03	[13]
17	TiN/SiO_2_	2	[44]
18	TiN/AlCrN	2.4	[28]
19	ZrO_2_/Y_2_O_3_	1.4	[7]
20	ZrO_2_:Y/SiO_2_	1.15	[7]
21	Si_3_N_4_/SiO_2_	1.14	[2]
22	HfO_2_/Al_2_O_3_	1.02	[9]
23	TiO_2_/Al_2_O_3_	0.95	[10]
24	Al_2_O_3_/SiO_2_	0.57	[45]
25	Cr_2_O_3_/SiO_2_	0.52	[45]
26	Y_2_O_3_/SiO_2_	0.5	[45]
27	Al_2_O_3_/SiO_2_	0.48	[2]
28	TiO_2_/SiO_2_ (this work)	0.21	

## Data Availability

The experimental ITR dataset and descriptor dataset can be found in the file “training dataset for ITR prediction” at https://doi.org/10.5281/zenodo.3564173 (accessed on 12 December 2022), which can be used as a training dataset for predicting ITR directly.
